# Levamisole Modulation of Podocytes’ Actin Cytoskeleton in Nephrotic Syndrome

**DOI:** 10.3390/biomedicines11113039

**Published:** 2023-11-13

**Authors:** Susan T. Veissi, Tijmen van den Berge, Joanna A. E. van Wijk, Thea van der Velden, René Classens, Lynn Lunsonga, Rick Brockotter, Charlotte Kaffa, Sander Bervoets, Bart Smeets, Lambertus P. W. J. van den Heuvel, Michiel F. Schreuder

**Affiliations:** 1 Department of Pediatric Nephrology, Amalia Children’s Hospital, Radboud Institute for Molecular Life Sciences, Radboud University Medical Center, 6525 GA Nijmegen, The Netherlands; 2 Department of Nephrology, Radboud Institute for Molecular Life Sciences, Radboud University Medical Center, 6525 GA Nijmegen, The Netherlands; 3 Department of Pathology, Radboud Institute for Molecular Life Sciences, Radboud University Medical Center, 6525 GA Nijmegen, The Netherlands; 4 Department of Pediatric Nephrology, Amsterdam University Medical Center, 1105 AZ Amsterdam, The Netherlands; 5 Center for Molecular and Biomolecular Informatics, Radboud University Medical Center, 6525 GA Nijmegen, The Netherlands; 6 Department of Laboratory Medicine, Radboud Institute for Molecular Life Sciences, Radboud University Medical Center, 6525 GA Nijmegen, The Netherlands; 7 Department of Development and Regeneration, University Hospital Leuven, 3000 Leuven, Belgium

**Keywords:** nephrotic syndrome, levamisole, podocyte, RNA sequencing, actin cytoskeleton

## Abstract

Podocytes play a central role in glomerular diseases such as (idiopathic) nephrotic syndrome (iNS). Glucocorticoids are the gold standard therapy for iNS. Nevertheless, frequent relapses are common. In children with iNS, steroid-sparing agents are used to avoid prolonged steroid use and reduce steroid toxicity. Levamisole is one of these steroid-sparing drugs and although clinical effectiveness has been demonstrated, the molecular mechanisms of how levamisole exerts its beneficial effects remains poorly studied. Apart from immunomodulatory capacities, nonimmunological effects of levamisole on podocytes have also been suggested. We aimed to elaborate on the effects of levamisole on human podocytes in iNS. RNA sequencing data from a human podocyte cell line treated with levamisole showed that levamisole modulates the expression of various genes involved in actin cytoskeleton stabilization and remodeling. Functional experiments showed that podocytes exposed to puromycin aminonucleoside (PAN), lipopolysaccharides (LPS), and NS patient plasma resulted in significant actin cytoskeleton derangement, reduced cell motility, and impaired cellular adhesion when compared to controls, effects that could be restored by levamisole. Mechanistic studies revealed that levamisole exerts its beneficial effects on podocytes by signaling through the glucocorticoid receptor and by regulating the activity of Rho GTPases. In summary, our data show that levamisole exerts beneficial effects on podocytes by stabilizing the actin cytoskeleton in a glucocorticoid receptor-dependent manner.

## 1. Introduction

Nephrotic syndrome (NS) is one of the most frequent glomerular diseases among children and is characterized by the triad of hypoproteinemia, massive proteinuria, and edema. NS may manifest secondary to other diseases, for instance, IgA vasculitis or lupus nephritis, but is most commonly named idiopathic (iNS) when no clear underlying condition has been identified [1]. The pathogenesis of iNS is thought to involve genetic alterations, immune dysregulation, and systemic circulating permeability factors (CPFs) that affect the functional integrity of podocytes [2]. Histological lesions in children with iNS mostly show minimal change disease (MCD, approximately 80%), followed by focal segmental glomeruloscerosis (FSGS) and membranoproliferative glomerulonephritis [3].

Corticosteroids represent the cornerstone for the management of iNS, and most children will show remission within four weeks of treatment with steroids [4]. However, the majority of iNS children will relapse within the first 6 months of initial therapy, and more than half will show frequent relapses and/or steroid dependency [3]. As chronic steroid use is associated with many side-effects, including weight gain, growth restriction, metabolic dysregulation, osteoporosis, striae and mood disorders, steroid-sparing immunosuppressive therapy is recommended in these patients [4]. Such agents may include cyclophosphamide, cyclosporin, tacrolimus, mycophenolate mofetil (MMF), levamisole, and rituximab [5]. Although their efficacy in iNS is not a subject of debate, all agents come at the cost of side effects—such as myelosuppression and nephrotoxicity—which often hampers their use in the pediatric population.

Levamisole is an anthelminthic drug with immunostimulatory properties and is also used as steroid-sparing agent in iNS [6]. Of all the aforementioned therapies, levamisole is considered the least toxic and the least expensive. Randomized clinical trials show that levamisole has a relative relapse risk reduction of 0.60 (95% confidence interval 0.45–0.79) and is well tolerated when administered at a dose of 2.5 mg/kg every other day [7]. Although its clinical efficacy is well established, there is a lack of molecular understanding of how levamisole exerts its beneficial effect in iNS. Apart from its immunostimulatory properties, it was recently shown that levamisole can also directly act on podocytes through the glucocorticoid receptor (GR) [8].

In the current study, we use an in vitro model of cultured conditionally immortalized human podocytes and plasmapheresis (PP) samples (as a source of CPFs) of selected iNS patients with active disease. With this model, we recently showed that CPFs cause oxidative stress and actin cytoskeleton rearrangement in podocytes, both of which are considered hallmark events in the pathogenesis of NS [9]. Using RNA sequencing, we demonstrate in the current study that levamisole alters the expression of many actin-binding and actin-remodeling proteins and functionally show that levamisole prevents actin cytoskeleton damage through GR activation and probably through subsequent actin regulating guanine triphosphate (GTPase) RhoA upregulation in podocytes exposed to NS patient plasma.

## 2. Materials and Methods

### 2.1. Patient Material

This study was conducted in accordance with the recommendations of the appropriate version of the Helsinki Declaration. Informed consent from all subjects was obtained. Patient samples were obtained as described previously [9]. Briefly, NS samples termed “active” were the first 500 mL of plasmapheresis (PP) samples from patients with steroid-resistant NS who required PP when immunosuppressive therapy alone (calcineurin inhibitor with or without the addition of rituximab) failed to induce remission. The remission plasma is the PP sample of the final PP session from a patient who was responsive to PP treatment. PP was initiated based on the decision from the treating physician using the Prismaflex system (Baxter International; Deerfield, IL, USA) with a TPE1000 or a TPE2000 filter, based on the size of the patient. All patient characteristics are listed in Table 1. Healthy control pool plasma was obtained from eight healthy adult volunteers. Exclusion criteria for this control group included fever, bacterial/viral infection in the past two weeks, chronic illness, inborn or acquired immune disorders, and the use of immunosuppressive drugs.

### 2.2. Cell Culture and Levamisole Treatment

Conditionally immortalized human podocytes (AB 8/13; kindly provided by Prof. Moin Saleem) were cultured in complete medium (CM; RPMI-1640 medium (Sigma, Zwijndrecht, The Netherlands), supplemented with 10% fetal bovine serum (FBS; Gibco, Thermo Fisher, Breda, The Netherlands), 1% insulin-transferrin-sodium selenite (ITS; Sigma, Zwijndrecht, The Netherlands), and 1% penicillin-streptomycin (Gibco, Thermo Fisher, Breda, The Netherlands)) at 33 °C with 5% CO_2_ under growth-permissive conditions. For experiments, podocytes were cultured at 37 °C with 5% CO_2_ under growth-restrictive conditions for 7 to 10 days in CM in order to differentiate. Differentiated podocytes seeded on 6-well plates (75,000 cells/well; Corning Costar^®^, Thermo Fisher, Breda, The Netherlands) were cultured in CM (vehicle; experimental replicate of *n* = 3 with three different passages of cells) or treated with 1 µM levamisole (experimental replicate of *n* = 3 with three different passages of cells; Sigma) for 24 h, and then subsequently subjected to RNA extraction and sequencing. 

### 2.3. RNA Extraction, cDNA Library Construction, and Sequencing

Bulk RNA sequencing was performed at Single Cell Discoveries (SCD, Utrecht, The Netherlands) using an adapted version of the CEL-seq protocol. Briefly, podocyte cell pellets stored in TRIzol (Invitrogen, Thermo Fisher, Breda, The Netherlands) were shipped on dry ice to SCD. Using the standard TRIzol method, total RNA was extracted from control and levamisole-treated podocytes, which was further used for library preparation and sequencing. Next, mRNA was processed for sequencing according to an adapted version of the single-cell mRNA seq protocol of CEL-seq as described previously [10,11]. Shortly, barcoding of the samples was performed by unique CEL-seq primers whereafter reverse transcription and second-strand synthesis was performed to generate cDNA. The latter was subjected to an overnight in vitro transcription reaction to generate amplified RNA, from which sequencing libraries were prepared using Illumina Truseq small RNA primers. Next, paired-end sequencing of the DNA library was performed on an Illumina Nextseq™ 500, high output, with a 1 × 75 bp Illumina kit (R1: 26 cycles, index read: 6 cycles, R2: 60 cycles). Read 1 was used to identify the Illumina library index and CEL-Seq sample barcode. Read 2 was aligned to the *Human Hg38* reference transcriptome using BWA MEM [12]. Reads that mapped equally well to multiple locations were discarded. Mapping and generation of count tables were performed using the MapAndGo script1. Normalization of the samples was conducted by RPM normalization. 

### 2.4. RNA Sequencing Data Analysis

RNA sequencing data were used to investigate the effects of levamisole on podocytes’ actin cytoskeleton-related genes. The following analysis steps were carried out to produce heatmaps of the differentially expressed genes: low-quality filtering and adapter trimming was performed using Trim Galore! v0.4.5 (Babraham Bioinformatics, Babraham, UK), a wrapper tool around the tools Cutadapt v1.18 and FastQC v0.11.8 (Babraham Bioinformatics). Reads were mapped to a human reference genome (GRCh38.95, Ensembl) with Star v2.7.5a, resulting in BAM files. BAM files were counted (number of reads mapped to a feature, e.g., a gene) with HTSeq (HTSeq-count tool v0.11.0) with default parameters using a complementary gtf file, containing an annotation for GRCh38.95 (Ensembl). MultiQC (quality control) was used to combine results and quality checks of all the samples. Differential gene expression analysis was carried out with DESeq2 v1.22.0 in R v3.5.3, with an internal statistical and normalization method (i.e., correction for multiple testing with the Benjamini–Hochberg procedure) using a cutoff value of at least 5 reads per sample per gene. Pathway and GO term enrichment were performed with selected genes based on *p*-value < 0.05.

### 2.5. Immunofluorescence Imaging

Podocytes (10,000 cells/well) were seeded on black 96-well tissue culture plate (Greiner Bio-One, Biosigma, Italy) and grown at 33 °C with 5% CO_2_ until 80% confluency, whereafter cells were transferred to 37 °C with 5% CO_2_ for differentiation. After 7–10 days, podocytes were stimulated with Lipopolysaccharide (LPS; O111) 20 µg/mL, puromycin aminonucleoside (PAN) 60 µg/mL, NS plasma, or control plasma (8% supplemented with FBS to retrieve 10% plasma [9]) in the presence or absence of GR inhibitor RU486 for 6 h followed by levamisole treatment (1 µM) for 24 h. Next, cells were fixed and stained as previously described [9]. Images were obtained with a Zeiss LSM880 laser scanning microscope (Zeiss, Oberkochen, Germany) using standard filter sets. 

### 2.6. Immunofluorescence Imaging Analysis

Fluorescence images obtained from phalloidin cytoskeletal staining experiments were analyzed using custom-made macros in the free open-source software Fiji/ImageJ v1.53t (available at the Zenodo data archive). First, conditionally immortalized human podocytes were manually outlined in order to perform single-cell measurements. Second, cellular phalloidin area and intensity were automatically determined using a readily available Fiji threshold algorithm. Third, phalloidin intensity under the nucleus was measured using DAPI as a selection, again using a readily available Fiji threshold algorithm. The same phalloidin and DAPI area selection algorithms were used for all raw images. As an internal quality control for automatic threshold selection, images containing the respective region of interest (ROI) selections (cellular area, phalloidin area, and nuclear area) were automatically created for post-processing analysis to ensure correct ROI selection in all conditions. 

### 2.7. Wound Closure Assay

Podocyte motility was assessed by a scratch-wound closure assay. Podocytes were seeded on 24-well plates (Corning Costar^®^, Thermo Fisher, Breda, The Netherlands) at a density of approximately 0.15 × 10^6^/well and then grown until 80% confluency, whereafter the cells were differentiated at non-permissive temperature for 7–10 days. Using a sterile 200 µL pipette tip, a scratch was applied to each well. Subsequently, podocytes were washed with phosphate-buffered saline (PBS) and exposed to plasma samples (8%) with or without levamisole (1 µM) during a time-lapse measurement of 24 h. Data were obtained with a Zeiss Axiovert 200M live cell imaging microscopy with a Moticam-pro 2850 CCD Camera. Data processing was performed at an interval of 15 min using Micromanager 1.4 software. Images were analyzed with Fiji/ImageJ v1.53t in which the total surface area of the scratched area (‘wound’) was calculated.

### 2.8. Cell Adhesion Assay

Podocytes were seeded on 12-well plates (Corning Costar^®^, Thermo Fisher, Breda, The Netherlands) and cultured at 33 °C with 5% CO_2_ until 80% confluency. After differentiation at 37 °C with 5% CO_2_, cells were stimulated with plasma samples (8%) with or without levamisole (1 µM) for 24 h. Next, cells were detached using Trypsin/EDTA (Gibco, Thermo Fisher, Breda, The Netherlands) and then centrifuged. Subsequently, cells were resuspended in 100 µL CM and then counted. Approximately 10,000 cells/well were seeded on 48-well plates (Corning Costar^®^, Thermo Fisher, Breda, The Netherlands) and allowed to adhere for one hour at 37 °C with 5% CO_2_. Non-adherent cells were washed using PBS (Gibco) and adhered cells were cultured in CM with CCK8 cell counting solution (Sigma) in order to determine the adhered podocytes [13]. Absorbance was measured at 450 nm with the Spark Multilabel Plate reader (Tecan, Giessen, The Netherlands).

### 2.9. Western Blotting

For protein extraction experiments, podocytes were seeded on 6-well plates (Corning Costar^®^) and grown at 33 °C with 5% CO_2_ until 80% confluency and then, as indicated previously, the cells were differentiated for 7 to 10 days at 37 °C with 5% CO_2_. For the GR experiments, podocytes were serum-starved for four hours in serum-free medium followed by levamisole (1 µM) treatment for 15, 30, and 60 min. For the GTPase experiments, podocytes were first stimulated with patient samples (8%) for 6 h followed by 24 h levamisole treatment. Total protein was extracted with the NucleoSpin^®^ RNA/Protein purification kit (Macherey-Nagel, Leiden, The Netherlands) according to the manufacturer’s protocol. Protein concentration was determined using the BCA Protein Assay kit (Pierce, Thermo Fisher, Breda, The Netherlands) according to the manufacturer’s protocol. Equal amount of protein (10 µg) under reducing conditions was used for sodium dodecyl sulfate polyacrylamide gel electrophoresis (SDS-PAGE; 10% resolving gel and 4% stacking gel) analysis. Next, protein was transferred onto polyvinylidene difluoride membranes (Merck, Zwijndrecht, The Netherlands) and blocked with PBS-5% bovine serum albumin (BSA) skim milk (Merck, Zwijndrecht, The Netherlands) for one hour at room temperature (RT). The membranes were washed with PBS-0.05% Tween20 (3×), followed by overnight incubation with primary antibodies (Abs), anti-glucocorticoid receptor (D6H2L), rabbit monoclonal Ab (mAb; 1 µg/mL; cell signaling technology, Leiden, The Netherlands), anti-phospho-glucocorticoid receptor (S134), Rabbit mAb (1 µg/mL; cell signaling technology, Leiden, The Netherlands), anti-mineralocorticoid receptor (Anti-NR3C2) rabbit polyclonal antibody (pAb; 1 µg/mL; Sigma), and anti-β-actin mAb (15G5A11/E2; 0.1 µg/mL; Invitrogen) diluted in PBS-5% BSA. Proteins of interest were detected with 0.25 µg/mL HRP-conjugated goat-anti rabbit or goat anti-mouse IgG (Dako, Leuven, Belgium) diluted in PBS-5% BSA for 1 h at RT. Rho GTPase activity was determined using an antibody kit according to the manufacturer’s protocol (9968T cell signaling technology). Chemiluminescence signal was detected either with Pierce™ ECL Western Blotting Substrate or SuperSignal™ West Pico PLUS Chemiluminescent Substrate (Thermofisher scientific, Breda, The Netherlands) and then visualized using Biorad Gel Doc XR Imaging System. Semiquantitative analysis of the blots was performed with ImageLab software v5.2.1. 

### 2.10. Statistics

All experiments were carried out as a minimum of *n* = 3 on three different passages of cells. Due to the limited amount of patient plasma, cell motility and cell adhesion experiments were performed *n* = 1 per patient sample and data of three active phase samples were pooled and expressed as a *n* = 3 representative of NS patients. All descriptive graphs and statistics were performed using GraphPad Prism software version 9. One-way ANOVA and Bonferroni post-hoc test analyses were used to identify statical significance if applicable. *p*-values less than 0.05 were considered statistically significant and 95% confidence intervals were reported when applicable. Statistical significance was reported in figures with use of these signs and correspond to *p*-values: * *p* < 0.05, ** *p* < 0.01, *** *p* < 0.001.

## 3. Results

### 3.1. Levamisole Modulates Genes Related to the Actin Cytoskeleton

We initially performed RNA sequencing of conditionally immortalized human podocytes treated with levamisole or vehicle, with the aim of uncovering the impact of levamisole on podocyte signaling pathways and processes. 

Following the initial analysis, we performed a differential gene expression (DEG) analysis and applied the Benjamini–Hochberg procedure. This comprehensive analysis revealed a total of 973 genes that were upregulated and 681 genes that were downregulated, all exhibiting a fold change greater than 2 or less than −2. We have presented a select subset of these genes in Supplementary Table S1. This focused selection comprises genes that demonstrated statistically significant changes, with a fold change exceeding 2 or −2. Specifically, our analysis identified 26 upregulated genes and 18 downregulated genes that met these stringent criteria.

**Figure 1:**
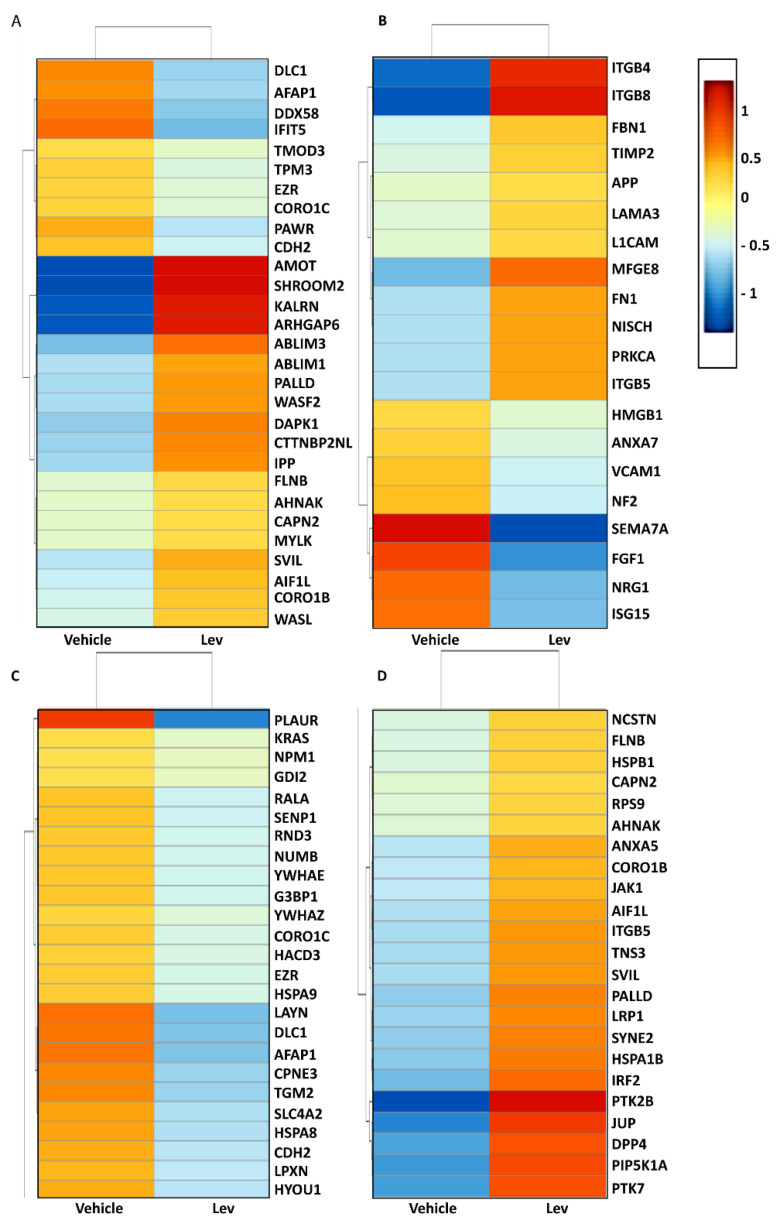
Levamisole seems to regulate actin cytoskeleton related genes in cultured human podocytes. Heat map of RNA seq expression data showing the genes associated with podocytes’ actin cytoskeleton (**A**), integrin binding proteins (**B**), and focal adhesion (**C**,**D**), which were differentially regulated following treatment with 1 µM levamisole for 24 h. Differentially expressed genes were selected based on a cut off value of at least 5 counts per sample per gene. Pathway and GO term enrichment were performed with selected genes based on *p*-value < 0.05.

Notably, as depicted in Figure 1, we observed significant modulation of numerous genes, either upregulated or downregulated, all of which are closely associated with specific cellular processes: actin cytoskeleton stabilization (Figure 1A), integrin binding (Figure 1B), and focal adhesions (Figure 1C,D).

Among the significantly upregulated genes, the most remarkable increases were observed in *AMOT* (*angiomotin*), *SHROOM2* (*Shroom family member 2*), *KALRN* (*kalirin*), and *ARHGAP6* (*Rho GTPase activating protein 6*), exhibiting fold changes of 2.26, 2.11, 1.38, and 1.58, respectively. These genes are particularly relevant to the regulation of the actin cytoskeleton.

These findings led us to hypothesize that levamisole functions as a stabilizer of the podocyte’s actin cytoskeleton.

### 3.2. Levamisole Prevents Actin Cytoskeleton Damage in a GR-Dependent Manner

**Figure 2:**
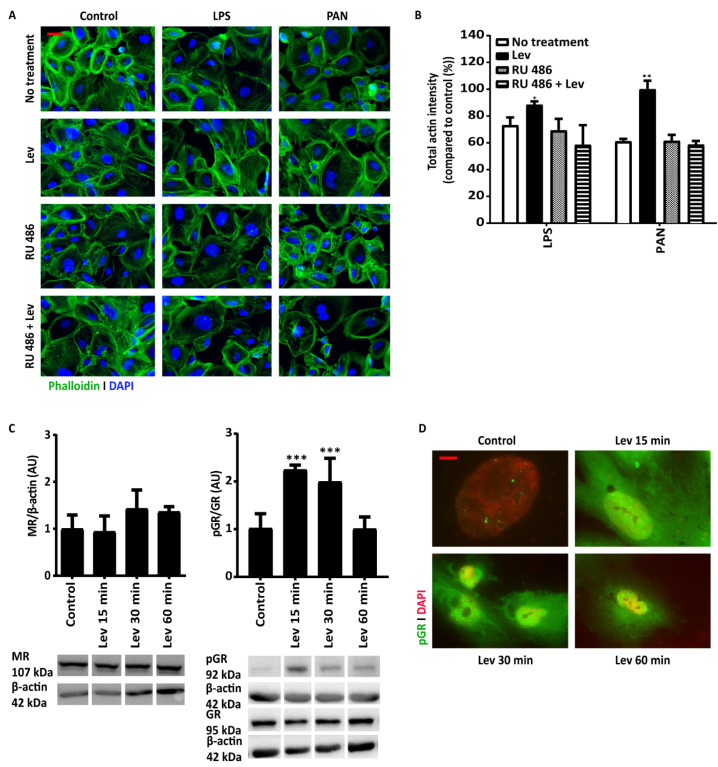
Levamisole signaling through the glucocorticoid receptor (GR). Levamisole treatment of podocytes (24 h) prevents podocytes’ actin cytoskeleton damage caused by LPS 20 µg/mL (O111) or PAN 60 µg/mL for 6 h. The pre-treatment of the cells with GR antagonist RU486 (1 µM for 1 h) seems to inhibit the protective role of levamisole (**A**). Quantification of the images confirms aforementioned observations (**B**). Levamisole treatment of podocytes induces significant upregulation of phosphorylated GR protein expression but not MR (**C**). Immunofluorescence images showing that levamisole (1 µM) treatment leads to GR phosphorylation and accumulation in the nucleus after 15 min (**D**). * *p* < 0.05, ** *p* < 0.01, *** *p* < 0.001 compared to the no treatment. Abbreviations: Lev, levamisole; LPS, lipopolysaccharide; PAN, puromycin aminonucleoside; MR, mineralocorticoid receptor; pGR, phosphorylated glucocorticoid receptor. Scale bars: 10 µm. All of the experiments were carried out as *n* = 3 with three different passages of cells.

To further test the hypothesis that levamisole acts as an actin stabilizer, we exposed podocytes to the injury inducers PAN and LPS and visualized whether the co-presence of levamisole could prevent damage to the actin cytoskeleton. As depicted in Figure 2A,B, podocytes stimulated with LPS and PAN exhibited a reduction in actin signal to 72% (SEM 5.9) and 61% (SEM 1.9) when compared to the control, respectively. Subsequent treatment of the cells with levamisole resulted in the restoration of the actin cytoskeleton to 88.2% (SEM 2.7) and 99.8% (SEM 6.5) in LPS and PAN conditions, respectively. We also included conditions with RU 486, a known inhibitor of GR, which prevented levamisole’s action to prevent actin loss upon exposure to either LPS or PAN (Figure 2A,B). Levamisole was shown to significantly increase phosphorylated GR protein exposure after 15 and 30 min, but not mineralocorticoid receptor (MR) expression (Figure 2C). GR is known to be activated by phosphorylation and subsequently translocates into the cell nucleus. This is further supported by immunofluorescence imaging of podocytes treated with levamisole for 15 and 30 min, which revealed an increased signal for phosphorylated GR, with rapid translocation into podocytic nuclei (Figure 2D). Together, these data suggest that levamisole functions as an actin stabilizer by signaling through the glucocorticoid receptor (GR).

### 3.3. Levamisole Prevents Actin Cytoskeleton Damage in Response to Active NS Plasma

**Figure 3:**
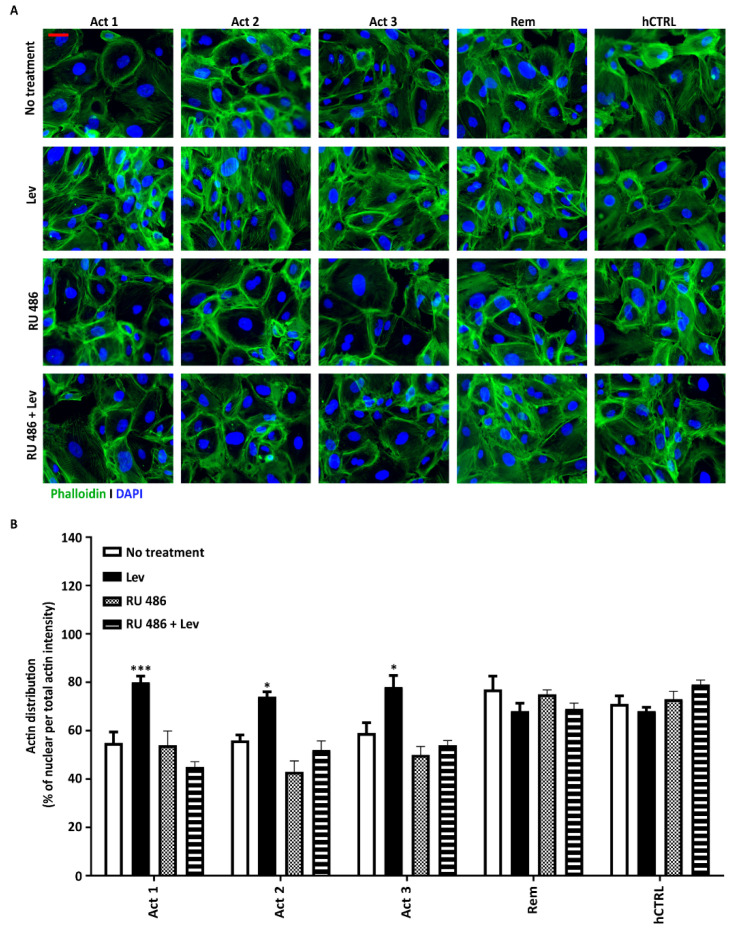
Levamisole ameliorates NS patient plasma-induced actin cytoskeleton damage in podocytes. (**A**) Podocytes stimulated with NS plasma (8%) for 6 h show significant damage, which can be prevented by post-treatment of the cells with levamisole (1 µM for 24 h). Pre-treatment of the cells with the GR inhibitor RU 486 (1 µM for 1 h) inhibits the action of levamisole (**A**). Immunofluorescence images quantified with Fiji confirmed above mentioned observations (**B**). * *p* < 0.05, *** *p* < 0.001 compared to the no treatment. Abbreviations: Lev, levamisole; Act 1, 2, and 3, active 1, 2, and 3; Rem, remission; hCTRL, healthy control pooled plasma. Scale bars: 10 µm. All of the experiments were carried out as *n* = 3 with three different passages of cells.

In addition to LPS and PAN, we also used plasma from active NS patients as a more biologically relevant stimulus. Plasma of active NS patients obtained during PP serves as a source of pathogenic CPFs [9]. Samples from (pooled) healthy controls or NS patients at remission were included as well. Patient characteristics are presented in Table 1. Even more prominently than LPS and PAN, the active NS plasma induced significant actin cytoskeleton derangement, resulting in reductions of 55% (SEM 4.49), 56% (SEM 2.3), and 59% (SEM 4.3) by Act 1, Act 2, and Act 3 plasma, respectively (Figure 3A,B). These effects were effectively mitigated by levamisole, leading to actin cytoskeleton restoration to 80% (SEM 2.5), 74% (SEM 1.97), and 78% (SEM 4.6) for Act 1, Act 2, and Act 3 plasma, respectively (Figure 3A,B).

### 3.4. Levamisole Improves Podocyte Motility and Adhesion in Response to NS Plasma

**Figure 4:**
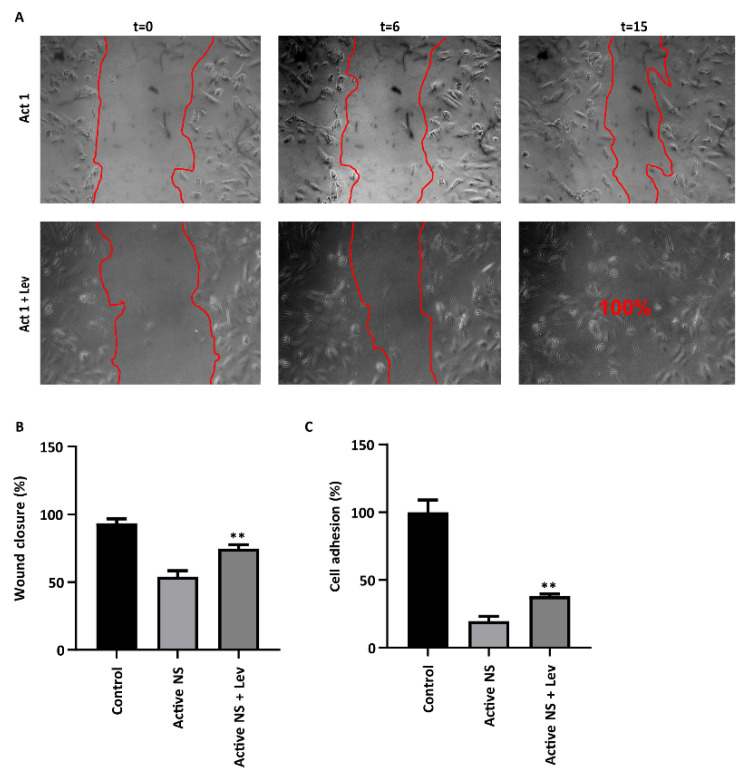
Levamisole abolishes NS plasma-induced damaged cell migration and adhesion. (**A**) Representative images from scratch assay showing the wound closure in podocytes stimulated with NS active 1 plasma with and without levamisole 1 µM at timepoints 0 h, 6 h, and 15 h. (**B**) Quantification of the data show that levamisole significantly improves the wound closure in podocytes stimulated with active NS plasma. (**C**) Levamisole treatment significantly improves cell adhesion in podocytes treated with active NS plasma (8%) for 24 h. ** *p* < 0.01 compared to the control. Abbreviations: Lev, levamisole; Act 1, active 1.

To functionally characterize the effects of levamisole, we performed wound closure and CCK8 assays to evaluate podocyte motility and adhesion, respectively. Both podocyte motility and adhesion are strongly dependent on the actin cytoskeleton. Wound closure was significantly impaired in response to all three active NS plasma, but the co-presence of levamisole improved wound closing capacities (Figure 4A,B). Similarly, podocyte adhesion was also impaired by active NS plasma, which could partially be abolished by the co-presence of levamisole (Figure 4C). These data imply that levamisole restores the deleterious effects of CPFs on the actin cytoskeleton in patients with active NS.

### 3.5. Levamisole Activates Rho A GTPases in Cultured Podocytes

**Figure 5:**
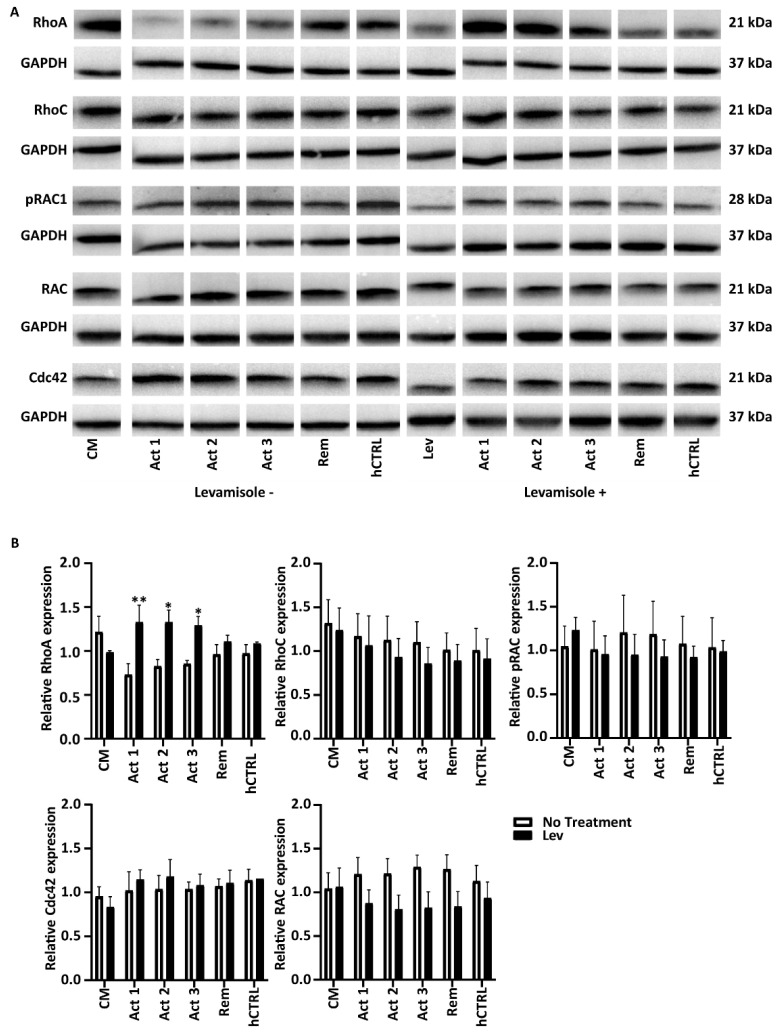
Levamisole seems to induce Rho A GTPase activation in podocytes in vitro. (**A**) Representative western blot images showing that podocytes exposed to active NS plasma (8%) for 8 h seems to result in decreased Rho A protein expression, which is again increased after post-treatment of the cells with levamisole (**B**). All of the experiments were carried out as *n* = 3 with three different passages of cells. * *p* < 0.05, ** *p* < 0.01.

Members of the rho family of GTPases have emerged as key regulators of the actin cytoskeleton. To obtain an indication of how levamisole may prevent actin cytoskeleton damage in cultured podocytes, we looked into the expression of the actin-related GTPases RhoA, Rac 1, RhoC, and Cdc42 by western blot (Figure 5). We observed that podocytes stimulated with NS plasma showed decreased expression of Rho A, which is significantly increased after treatment with levamisole (Figure 5A,B). Differences in Rac 1, RhoC, and Cdc42 expression between conditions with and without levamisole were observed as well, although significance could not be reached by quantification. 

## 4. Discussion

The immunomodulatory properties of levamisole have long been considered key in its capacity to prevent relapses of iNS in children. Previous studies showed that levamisole activates dendritic cells and enhances T-cell activation towards a type 1 T helper immune response [14,15]. We now show that levamisole also has a direct effect on podocytes, whose aberrant functioning is a pathogenic hallmark of iNS.

The concept that immunosuppressive therapy may be beneficial for iNS patients because of nonimmunological mechanisms of renoprotection is increasingly acknowledged [16]. For instance, Faul et al. demonstrated the prevention of synaptopodin degradation by cathepsin L and the subsequent stabilization of the actin cytoskeleton in podocytes treated with calcineurin inhibitors [17]. Another example is the direct action of rituximab on sphingomyelin-phosphodiesterase-acid-like-3b (SMPDL-3b) expression in podocytes, whose upregulation by rituximab can prevent disruption of the actin cytoskeleton in response to FSGS sera [18]. Finally, the increase in Rho A activity in podocytes treated with steroids appears pivotal for actin stabilization and podocyte functioning [19]. These observations taken together suggest that the podocyte-protecting capacity of these commonly used agents seems to lie (at least partly) in the stabilization of the actin cytoskeleton. 

The podocyte’s actin cytoskeleton is crucial for its stability, dynamic response to environmental stimuli, slit diaphragm insertion, and adherence to the glomerular basement membrane [20]. Podocyte injury in NS is thought to be initiated by derangement of the actin cytoskeleton, which ultimately leads to characteristic foot process effacement and/or podocyte detachment [21]. We recently showed that PP samples of active NS patients cause actin cytoskeleton damage to cultured podocytes [9]. In the current study, we demonstrate that podocytes stimulated with PAN and LPS show a significant decrease in total actin signal whereas podocytes exposed to NS plasma did not differ in terms of total actin signal intensity yet resulted in significant derangement of the actin cytoskeleton. We show that damage to the actin cytoskeleton in response to these samples can be attenuated by the post-treatment with levamisole for 24 h. Interestingly, the simultaneous blocking of GR with RU 486 (mifepristone) prevented this action of levamisole, suggesting that levamisole signals through GR, an observation that is in line with previous reports [8]. Activation of GR by steroids was previously shown to cause actin filament stabilization in murine podocytes [22]. RNA sequencing data from dexamethasone-treated human podocytes showed similarly to our result, significant upregulation of genes that are important for the functionality of the actin cytoskeleton processes [23]. Levamisole thus seems to mimic the actions of glucocorticoids, which may explain its steroid-sparing effect. 

How GR signaling prevents actin cytoskeleton damage in podocytes remains not completely clear. One plausible explanation lies in the modulating effects of GR signaling on oxidative stress [24,25]. We previously found that actin cytoskeleton damage in response to NS plasma could be prevented by inhibition of reactive oxygen species (ROS) formation [9]. The observation that levamisole reduces the expression of the ROS inducers Nox4, p22phox, and p47phox in podocytes treated with PAN is in line with this concept [8]. Furthermore, the RNA sequencing data from levamisole-treated podocytes also showed expression of genes such as *SESN2*, *TP53*, and *EROA1*; all are involved in the cellular response to stress [26,27]. Another explanation is that GR is a transcription factor for many genes—amongst others, those involved in actin cytoskeleton stabilization, such as rho GTPases [28,29]. Indeed, we found that podocytes treated with levamisole induced GR phosphorylation and trafficking towards the nucleus, which is thought to be associated with activation of the GR [30]. We also observed that levamisole regulated Rho GTPase activity in podocytes. In addition to rho GTPases, transcription targets of GR also include *AMOT*, *SHROOM2*, *KALRN*, and *ARHGAP6*; all of these actin-stabilizing genes were significantly upregulated in levamisole-treated podocytes, as our RNA sequencing data revealed. 

Of note, we show that levamisole is able to ameliorate podocyte actin cytoskeleton damage not only in podocytes exposed to PAN and LPS, but also in podocytes stimulated with plasma of iNS patients. One of the major limitations of our study is the limited number of NS patient plasmas used, even though the results from the PAN, LPS, and PP studies were similar in effect. Unfortunately, from a research point of view, few patients will show a relapse of FSGS after kidney transplantation, and from these patients, plasma samples are rarely stored. To enable reproduction and validation of our results with larger patient cohorts, international collaborations are needed. Furthermore, our observations are based on in vitro cultured podocyte experiments. For many experiments, models that more closely mimic the in vivo situation, such as co-culture models including mesangial and endothelial cells or, alternatively, the use of kidney organoids, are preferred over the “old-fashioned” culture of a single layer of cells [31]. However, the 2D culture of immortalized podocytes allows for the optimal assessment of the actin cytoskeleton architecture, which was the main focus of our experiments. Confirming our findings in vivo should also be the focus of ongoing research. A recent study by Suleiman et al. applied intriguing super-resolution microscopy techniques that are able to visualize actin cytoskeleton reorganization in vivo; utilization of this technique in the context of iNS would be of great value to further strengthen the observations made in our study [32].

In conclusion, our data provide further mechanistic insight in the renoprotective effects of levamisole by targeting the GR–actin axis. The targeting of proteins or pathways involved in actin cytoskeleton stabilization appears to be a shared immune-independent mechanism of immunosuppressive agents that are currently used in iNS. These observations call for a search for new drugs specifically targeting actin and that do not come with the adverse reactions associated with immunosuppression.

## Figures and Tables

**Table 1:** Patient characteristics.

Sample	Sex	Age	eGFR (mL/min/1.73 m^2^)	P/C Ratio (g/10 mmol)	Albumin (g/L)	Oedema	Transplant	Diagnosis
Rem	M	16	73	0.61	28	None	No	FSGS, in remission
Act 1	F	7	77	19	20	Some	Yes	FSGS, relapsing
Act 2	M	15	34	8.83	23	Some	No	FSGS, first onset
Act 3	M	4	18	15	19	Severe	No	FSGS, first onset

Abbreviations: Rem, remission; Act 1/2/3, active 1/2/3; eGFR, estimated glomerular filtration rate; F, female; M, male; FSGS, focal segmental glomerulosclerosis; P/C ratio, urinary protein-creatinine ratio.

## Data Availability

Bulk RNA sequencing data has been deposited in Gene Expression Omnibus under accession number GSE229250. The custom-made Fiji macro used for phalloidin cytoskeletal staining analysis are available on Zenodo: https://doi.org/10.5281/zenodo.7711830.

## Supplementary Materials

The following supporting information can be downloaded at: https://www.mdpi.com/article/10.3390/biomedicines11113039/s1. Table S1. Upregulated genes. Table S2. Downregulated genes.
