# Supplementary material for: Levamisole Modulation of Podocytes’ Actin Cytoskeleton in Nephrotic Syndrome
Source: Biomedicines. 2023 Nov 13;11(11):3039. doi: 10.3390/biomedicines11113039 (PMC10669662; doi:10.3390/biomedicines11113039)
Supplement: Supplementary file 1 [file biomedicines-11-03039-s001.zip › biomedicines-2610794-supplementary.pdf]

# Levamisole Modulation of Podocytes' Actin Cytoskeleton in Nephrotic Syndrome

**Susan T. Veissi [1], Tijmen van den Berge [2,3], Joanna A. E. van Wijk [4], Thea van der Velden [1], René Classens [1], Lynn Lunsonga [1], Rick Brockotter [1], Charlotte Kaffa [5], Sander Bervoets [5], Bart Smeets [3,†], Lambertus P. W. J. van den Heuvel [1,6,7,†] and Michiel F. Schreuder [1,*,†]**

1 Department of Pediatric Nephrology, Amalia Children's Hospital, Radboud Institute for Molecular Life Sciences, Radboud University Medical Center, 6525 GA Nijmegen, The Netherlands; susan.veissi@radboudumc.nl (S.T.V.); thea.vandervelden@radboudumc.nl (T.v.d.V.); rene.classens@radboudumc.nl (R.C.); lynnluns@gmail.com (L.L.); rick.brockotter@radboudumc.nl (R.B.); bert.vandenheuvel@radboudumc.nl (L.P.W.J.v.d.H.)

2 Department of Nephrology, Radboud Institute for Molecular Life Sciences, Radboud University Medical Center, 6525 GA Nijmegen, The Netherlands; tijmen.vandenberge@radboudumc.nl

3 Department of Pathology, Radboud Institute for Molecular Life Sciences, Radboud University Medical Center, 6525 GA Nijmegen, The Netherlands; bart.smeets@radboudumc.nl

4 Department of Pediatric Nephrology, Amsterdam University Medical Center, 1105 AZ Amsterdam, The Netherlands; jae.vanwijk@amsterdamumc.nl

5 Center for Molecular and Biomolecular Informatics, Radboud University Medical Center, 6525 GA Nijmegen, The Netherlands; charlotte.kaffa@radboudumc.nl (C.K.); sander.bervoets@radboudumc.nl (S.B.)

6 Department of Laboratory Medicine, Radboud Institute for Molecular Life Sciences, Radboud University Medical Center, 6525 GA Nijmegen, The Netherlands

7 Department of Development and Regeneration, University Hospital Leuven, 3000 Leuven, Belgium

* Correspondence: michiel.schreuder@radboudumc.nl; Tel.: +31-24-36-14430

† These authors contributed equally to this work.

**Supplement Table S1**

**Table S1.** Upregulated genes

| Gene ID | $Log_2$ (Fold Change) | p-value |
|---|---|---|
| LRRN3 | 2,081724204 | 0,003812851 |
| OVGP1 | 2,403317845 | 0,004125599 |
| AC009948.1 | 4,257079279 | 0,00453766 |
| NMNAT3 | 2,378113699 | 0,010369072 |
| COL4A3 | 2,664064944 | 0,03204568 |
| RAB7B | 2,261612795 | 0,032242056 |
| AC025580.1 | 2,21853399 | 0,033849847 |
| AQP11 | 2,020752284 | 0,049478528 |
| ABCA1 | 2,143859504 | 0,050590857 |
| SCARNA9 | 2,262650654 | 0,055628269 |
| NGFR | 2,099464268 | 0,064908361 |
| H19 | 2,958833729 | 0,067622752 |
| LRRK2 | 2,044060798 | 0,070606901 |
| HLA-DMB | 3,206999816 | 0,071334989 |
| ENTPD3 | 2,178967212 | 0,077128476 |
| CYP1B1 | 2,123654638 | 0,088895028 |
| SOX6 | 2,258060036 | 0,092270592 |
| AC112484.3 | 2,021115784 | 0,096850265 |
| BLNK | 4,323374859 | 0,133212145 |
| CXCL12 | 2,479369747 | 0,152544086 |
| GAS7 | 2,18916781 | 0,170488639 |
| RHOJ | 2,196172101 | 0,174514138 |
| FREM2 | 2,013735805 | 0,18209847 |
| OLFML2B | 2,037960986 | 0,225773573 |
| SCD | 2,061276209 | 0,228338704 |
| KCNB2 | 2,237743209 | 0,258634597 |

Statistically significant genes exhibiting a 2-fold increase in expression in levamisole-treated podocytes, with fold change calculated relative to vehicle-treated cells (podocytes without treatment).

**Supplement Table S2**

**Table S2.** Downregulated genes

| Gene ID | $Log_2$ (Fold Change) | p-value |
|---|---|---|
| TRAF1 | -2,390997 | 0,000021 |
| ID3 | -2,800503 | 0,000022 |
| ID1 | -2,252142 | 0,001395 |
| NR4A1 | -2,789682 | 0,004191 |
| AL713998.1 | -2,153311 | 0,006656 |
| POU4F2 | -2,703443 | 0,009294 |
| RAB42 | -3,158638 | 0,012932 |
| ADD2 | -2,166787 | 0,014279 |
| IL11 | -2,172981 | 0,019970 |
| ANGPTL4 | -3,131751 | 0,022906 |
| PITX1 | -2,377637 | 0,028096 |
| BCL2A1 | -2,401348 | 0,030945 |
| HAS2 | -2,056177 | 0,042031 |
| NR4A3 | -3,265000 | 0,051889 |
| KRT81 | -2,340025 | 0,068464 |
| RPS3A | -2,747924 | 0,069245 |
| C15orf39 | -2,260437 | 0,070607 |
| CHGA | -2,086104 | 0,308686 |

Statistically significant genes exhibiting a 2-fold reduction in expression in levamisole-treated podocytes, with fold change calculated relative to vehicle-treated cells (podocytes without treatment).
